# Analysis of the Flow Behavior of Mechanically Fibrillated Cellulose Nanofibril Suspension by the Rheo-Polarized Imaging Technique (Rheo-Iris)

**DOI:** 10.1021/acs.langmuir.5c04628

**Published:** 2025-10-16

**Authors:** Moe Araida, Yoshifumi Yamagata, Saki Otobe, Yoshitaka Osafune, Taisuke Sato, Takuya Katashima, Gareth H. McKinley

**Affiliations:** † Anton Paar Japan K. K., First Fl., Reverside Sumida, 1-19-9Tsutsumi-dori, Sumida-ku, Tokyo 131-0034, Japan; ‡ Department of Bioengineering, Graduate School of Engineering Department of Bioengineering, 13143 The University of Tokyo, 7-3-1 Hongo, Bunkyo-ku, Tokyo 113-8656, Japan; § Research Institute for Science and Technology, Tokyo University of Science, 2641 Yamazaki, Noda-shi, Chiba 278-8510, Japan; ∥ Photonic Lattice, Inc., LABO CITY SENDAI, 6-6-3 Minami-Yoshinari, Aoba-Ku, Sendai-City, Miyagi 989-3204, Japan; ⊥ Department of Mechanical Engineering, Massachusetts Institute Technology. 77 Massachusetts Avenue, Room 3-254, Cambridge, Massachusetts 02139, United States

## Abstract

Understanding the coupling between the dynamics of microstructural deformation and the bulk flow behavior of colloidal suspensions is crucial for both fundamental studies and practical applications of this important class of soft matter systems. In this study, we investigated the flow behavior of cellulose nanofibril (CNF) suspensions—renewable, sustainable materials with low environmental impact—using the “Rheo-Iris,” a rheo-polarized imaging system we developed to visualize two-dimensional microstructural changes in fluid under applied stress. Creep tests under constant shear stress revealed an initial elastic material response, a yield transition, followed by viscoplastic flow at long times. Simultaneous polarized imaging identified three distinct retardation patterns, depending on the applied shear stress. At low stresses (≤10 Pa), or small strain immediately after stress application, the phase retardation remained uniformly low, and the orientation axis of the microstructure was randomly distributed, indicating that the homogeneously dispersed CNFs form an isotropic, entangled network structure. At a stress near the yield point (40 Pa), a spiral-shaped region of high retardation appeared, and the orientation axis shifted to 60–85° away from the flow direction. This corresponds to the formation of rosary-like structures aligned in the vorticity direction, exhibiting spatially nonuniform birefringence and an oriented microstructure. At higher stresses far above the yield point (200 Pa), this “log-rolling” mesostructure collapsed, and smaller CNF aggregates became aligned in the flow direction, leading to spatially uniform oriented birefringence across the entire field. Both cases represent distinct fiber orientation phenomena, and our noninvasive rheo-polarization method clearly distinguishes how the spatial orientation distribution in the field changes with applied stress. The Rheo-Iris system enables real-time, quantitative analysis of internal microstructural evolution under imposed shear strain or stress and offers a powerful tool for exploring the orientation dynamics in soft matter systems, opening a new eye on complex fluid rheology in colloidal dispersions.

## Graphical abstract

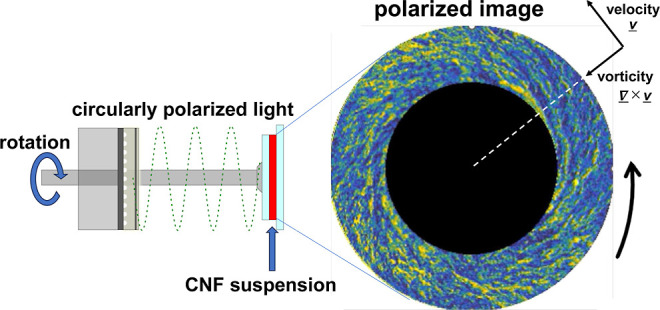

## 1 Introduction

Investigating the deformation and flow behavior of colloidal suspensions when subjected to external forces is an extremely important topic, not only for understanding the microstructural mechanisms that control their fundamental rheological behavior but also for tuning the material properties for practical applications. For these reasons, they have been intensively studied using a range of experimental and numerical methods from various perspectives. The flow properties of colloidal suspensions depend on such variables as the particle concentration, shape, interparticle interactions, and shear rate. For example, if the concentration of the dispersed phase is low and there is no interaction between the particles, the viscosity of the system is Newtonian and follows Einstein’s viscosity formula. However, as the concentration increases, interactions among the particles come into play, causing the system’s viscosity to rise rapidly and ultimately leading to gelation. In addition, when shear is applied, many suspensions exhibit pronounced shear-thinning behavior in which the apparent viscosity decreases. This decrease in viscosity can be interpreted as arising from the progressive shear-driven destruction of the aggregated microstructure formed by the electrostatic and depletion interactions between individual dispersed particles.^1–4^ On the other hand, shear-thickening behavior, in which the viscosity increases due to the reaggregation of dispersed particles caused by the rate of collisions between the clusters, has been observed in certain systems^5–7^ and this can lead to the formation of a shear-induced “shake gel”, in which gelation occurs by intense shaking.^8–11^

Therefore, to clarify the flow behavior exhibited by colloidal suspensions due to the applied shearing, it is important to dynamically and quantitatively evaluate how the microstructure formed by the dispersed particles breaks down and collapses under shear or how the dispersed particles form an increasingly aggregated structure under flowing conditions. The size of the dispersed particles and the scale of the aggregated meso-structures they form under flow also vary from nanometer to millimeter scale. Therefore, evaluating the evolution of the hierarchical microstructure at each scale is important, from orientation of the primary microstructural constituents to the higher-order mesoscale structure formed by aggregation.

Many attempts have been made to investigate the relationships between rheological properties and their molecular origins using combinations of bulk mechanical rheometry and spatially localized optical measurements (rheo-optics). Conventional rheo-optical measurements can be primarily classified into two classes: rheo-microscopy and rheo-scattering measurements. Rheo-microscopy directly observes the samples under shear flow using optical microscopes and confocal laser microscope techniques.^12–16^ For example, the transitional changes in the floc size of microfibril cellulose (MFC) in a MFC suspension have been directly observed using a CCD camera attached to a rotational rheometer equipped with a transparent coaxial double cylinder fixture.^12,13^ It has been reported that the floc size of MFC suspensions decreases with increasing shear rate, while the floc size progressively increases in the low shear rate range over which a plateau viscosity region appears. However, these measurements were analyzed from still images taken after the shear was stopped, and the floc size was not dynamically measured during shearing, because of the difficulty in obtaining a clear unblurred image during continuous shearing. In addition, observations with an optical microscope were used to study microparticles undergoing Brownian motion and suspensions flowing through microchannels. These methods mainly capture macroscopic features of the flow, and it is difficult to dynamically capture the processes of microstructural aggregation and destructuration experienced by dispersed particles, as the aggregates form and then collapse by the applied shearing. The biggest challenge in using a rheomicroscope is the limited field of view. In regions with high shear rates, focusing on multiple dispersed particles becomes difficult, and the restricted observation area limits the ability to obtain sufficient ensemble averaging. These limitations make it challenging to characterize dynamic behaviors under such conditions accurately.

On the other hand, scattering methods evaluate the flow behavior of colloidal suspensions from nano- to micron size in the Fourier domain using methods such as small-angle neutron scattering (SANS) and small-angle X-ray scattering (SAXS).^17–22^ Shibayama et al. carried out Rheo-SANS experiments on an inorganic clay mineral and polyethylene glycol system that exhibits a shear-thickening behavior and reported that anisotropy appears in the SANS scattering pattern in the shear rate regime corresponding to increasing viscosity.^17^ They proposed that the long PEO chains that have adsorbed on the individual clay platelets are progressively peeled off when sufficiently strong shearing is applied, but that they readsorb again and form interparticle cross-links with the neighboring clay platelets. However, the weak intensity of the SANS beam required 1–30 min of exposure to obtain a complete scattering spectrum. Therefore, the results are not time-resolved, and there is a possibility that the viscosity of the system will gradually change due to irreversible shear-induced damage to the sample during the measurement. Akada et al.^18^ used SAXS and ultrasmall angle X-ray scattering (USAXS), which is suitable for time-resolved measurements with short exposure times, to observe the change from a liquid to gel in a colloidal suspension comprised of silica particles and poly­(ethylene oxide) polymer chains in real-time. SAXS can evaluate structures on the order of tens of nanometers, while USAXS can evaluate structures on the order of hundreds of nanometers. According to their report, the system showed shear-thickening behavior at around 10 s^–1^, in which the intensity in the USAXS spectrum in the direction of the vorticity increased, although the scattering intensity obtained using SAXS did not change. These results suggest that there is no change in the distribution of the clusters of silica particles at the scale of several tens of nanometers; however, larger aggregates of several hundred nanometers formed by shear-induced aggregation are oriented in broadly elliptical clusters in the flow direction. All these studies examine the flow behavior of colloidal suspensions based on nanoscale microstructural changes.

In addition, other rheo-optical studies at the micron size have employed rheo-small angle light scattering (Rheo-SALS) measurements.^19–21^ Pignon et al. investigated the flow behavior of thixotropic clay suspensions and reported that the optical scattering resulting from the sample microstructure was isotropic in the rest state, but as the shear rate increased, the scattering pattern was enhanced in the direction of the velocity, and above a critical shear rate, the scattering pattern became isotropic again.^19^ They proposed the following interpretation of these results: at low shear rates, the dispersed network of clay platelets in the suspensions align and consolidate under the action of hydrodynamic forces, and the dispersed particles form a roller-shaped aggregate. These roller-shaped aggregates are aligned in the direction perpendicular to the flow direction (i.e., the vorticity direction) corresponding to a “log-rolling state”.^23–25^ Beyond a critical shear rate, no further consolidation is possible, and the roller-shaped aggregates are progressively broken down into smaller pieces with an increasing shear rate. Such an interpretation is consistent with theoretical studies performed on the disaggregation process during flow.^26,27^ The formation of an aggregated structure oriented in the vorticity direction of dispersed particles with the applied shearing has also been confirmed in a latex particle suspension containing associative polymers by Belzung et al.^20^ Furthermore, the orientation behavior in the vorticity direction has been observed not only in thixotropic suspensions but also in clay mineral-surfactant suspensions that exhibit shear-thickening behavior.^10^

It is expected that combining rheo-SALS, SAXS, and USAXS measurements will enable structural observation across multiple scales from the single constituent particle scale (primary structure) to that of the spatial organization of the aggregates (higher-order mesoscale structure). The integration of mechanical bulk rheometry with time-resolved scattering techniques offers a comprehensive view of the structural dynamics under various flow conditions, enhancing our understanding of the evolving microstructural dynamics and rheological response of complex materials.^28^ However, while these scattering techniques are pointwise measurements, the microstructural information obtained is limited to the local interrogation area through which the incident beam (such as the collimated light or X-ray source) is irradiated. This allows us to locally evaluate the ensemble average information, but spatial distribution of the microstructure on the scale of the flow remains inaccessible. Therefore, it is extremely important to perform two-dimensional time-resolved imaging to quantitatively evaluate the flow behavior of colloidal suspensions that form spatially heterogeneous structures (described in the Materials and Methods section). We have, therefore, used a new rheo-optical device that combines a parallel plate rheometer configuration with a high-speed polarization camera capable of two-dimensional dynamic polarization analysis to examine the flow behavior of suspensions of anisotropic colloidal particles and how the microstructure evolves with applied shearing.

Sato et al.^29^ employed stress ramp tests using a rheo-optical device equipped with a polarized high-speed camera to clarify the relationship between yield behavior and structural evolution in 2,2,6,6-tetramethylpiperidine-1-oxyl radical (TEMPO)-oxidized CNF suspensions. According to their report, a two-step yield behavior was observed in the stress ramp tests, and distributions of retardation and orientation axes showed characteristic patterns in three regions. However, spatial and temporal tracking of the retardation distribution in the vicinity of the yield transition, and quantitative analysis of the shear-induced changes in the microstructure, have not been investigated in detail. In the present work, we employ constant-stress (creep) using the aforementioned rheo-optical device to explore the microstructural evolution of a CNF suspension prepared by mechanical treatment. We follow the shear-induced changes in the internal structure of the sample by tracking the spatial and temporal changes in the retardation distribution under progressively increasing values of the imposed constant stress. Since the color annular images of the shear-induced microstructure in the colloidal suspensions resemble an iris, and the rheo-optical device combines a rheometer and a high-speed polarization camera, we refer to this device as a “Rheo-Iris” that opens a new eye on complex fluid rheology.

## 2 Materials and Methods

### 2.1 Sample

The CNF suspension is a commercially available product, and we used the extra-long type (IMa-10002) made by Sugino Machine Limited. The concentration of the CNF suspension was 2 wt %. This suspension was manufactured by mechanical fibrillation (water jet method) and used "as is" without any special treatments.

### 2.2 Measurement of the CNF Size

To measure the size of the CNFs, we used a SAXS device (SAXSpace, Anton Paar GmbH, Graz, Austria) and a dynamic image analysis device (Litesizer DIA 500, Anton Paar GmbH, Graz, Austria).

For the SAXS measurement, a Cu-Kα (wavelength λ = 0.154 nm) X-ray source and a capillary cell were used, and the measurement was performed at 25 °C. In the dynamic image analysis method, 2 mL of a CNF suspension was added to 18 mL of ultrapure water and dispersed for 10 min under 200 W ultrasound excitation. The ultrasonically dispersed sample was added in its entirety to a device tank filled with 600 mL of ultrapure water, and the measurement was repeated five times under 50 W ultrasound excitation.

### 2.3 Rheological Measurements

Rheological measurements were carried out using a stress-controlled rheometer (MCR302e, Anton Paar GmbH, Graz, Austria). Dynamic viscoelasticity measurements (discrete frequency sweeps) were performed by using parallel plate fixtures with a diameter of 40 mm and a 0.5 mm gap between them. The same configuration was used for strain amplitude measurements. For the creep tests, a quartz upper plate with a diameter of 43 mm was used, and the gap was again set to 0.5 mm. Stresses of 10, 40, 60, and 200 Pa were applied for 120 s, and the evolution in the resulting sample strain γ­(*t*;σ_0_) with time was measured. All rheological measurements were performed at 20 °C.

### 2.4 Rheo-Polarized Imaging (Rheo-PI) Measurement

Figure 1 shows a schematic diagram of the experimental setup (Rheo-Iris).^29^ This device is equipped with a high-speed polarization camera (CRYSTA PI-5WP; Photonic Lattice Inc., Japan) that can measure the spatial distribution of the optical retardation (birefringence) and orientation axes in real time from the underside of the stress-controlled rheometer. By combining the controlled stress rheometer with a high-speed polarization camera, it is possible to simultaneously measure the evolution of rheological properties and the microstructural dynamics of soft solids and complex fluid materials. The head of the rheometer is equipped with a light source that combines a ring LED and circular polarizing film. The circularly polarized light emitted from the light source is irradiated onto the sample, and the transmitted light is imaged by the high-speed camera at the bottom. For optical measurements, the information obtained about the internal microstructure of the sample differs, depending on the transmission direction of the light. For example, for simple shear flows (in which the 1-direction corresponds to the velocity (flow) direction, the 2-direction represents the velocity gradient direction and the 3-direction is the vorticity orientation), if the light propagates along the vorticity direction, structural information on the 1–2 surfaces can be obtained, and if the light is transmitted along the velocity gradient direction, information on the 1–3 shearing surfaces can be obtained. However, for the tests using plate–plate geometry, it is difficult to transmit light along the shear or vorticity directions. Therefore, in this study, we will consider changes in the internal structure of the sample based on imaging of the 1–3 shearing plate information obtained by transmitting light along the velocity gradient direction. The frame rate for the video recording was set to 30 fps.

**Figure 1:**
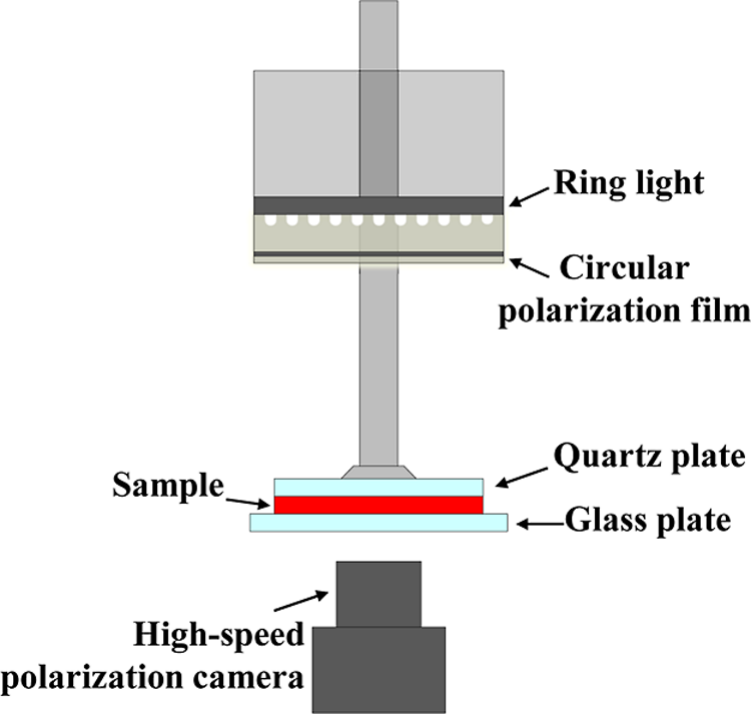
Schematic diagram of the Rheo-Iris device.

## 3 Results and Discussion

### 3.1 Size of CNFs

The SAXS measurements can evaluate the cross-sectional diameter of the CNFs. Figure 2a shows the scattering curve of a CNF suspension. The actual measured values are the black curve. On the other hand, when a fitting was performed, assuming the shape of the CNFs to be cylindrical (red dashed curve in Figure 2a), a good match was obtained with the actual measured values. To determine the fibril average cross-section diameter (2*r̅*), we conducted data evaluations based on the inverse Fourier transformation (IFT).^30–32^ The pair distribution function (PDF) obtained from the IFT method is shown in Figure 2b. As can be seen from the figure, the radius *r* of the CNF has a wide distribution of 10–60 nm with a mean value around *r̅* = 31 nm. Since it has been reported that the diameter of a single cellulose microfibril is about 3 nm,^33^ it is suggested that the CNFs used in this study exist in bundles consisting of tens of microfibrils.

**Figure 2:**
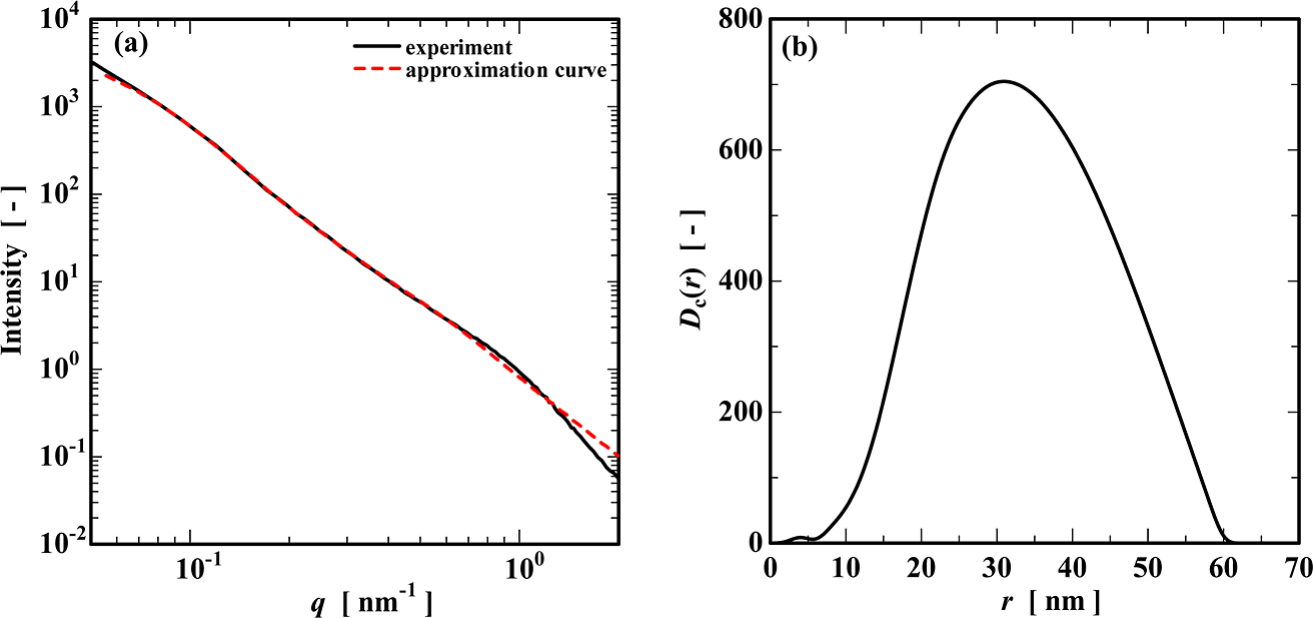
(a) SAXS profile and (b) diameter distribution of the CNFs.

In the dynamic imaging analysis (DIA), fibrils, such as the CNFs and particles, are directly photographed, and the size and shape can be analyzed by capturing the outline of the specimens.^34,35^ The scatter diagram of the diameter and length of the CNFs calculated by DIA measurements is shown in Figure 3, and the fibril diameter and length were distributed in the ranges of 2–6 and 4–100 μm, respectively. There is thus a significant difference in the range of fibril diameter determined by direct image analysis compared to the values obtained from the SAXS measurements. This is due to the difference in the scale of observation, with the SAXS and DIA measurements corresponding to analyses on the nanoscale and micron-scale ranges, respectively. In addition, since it can be assumed that the CNFs produced by the mechanical fibrillation process will show a hierarchical fibril diameter distribution, it is also possible that the CNFs used in this study have a bimodal distribution of diameters of 10–60 nm and 2–6 μm.^36^

**Figure 3:**
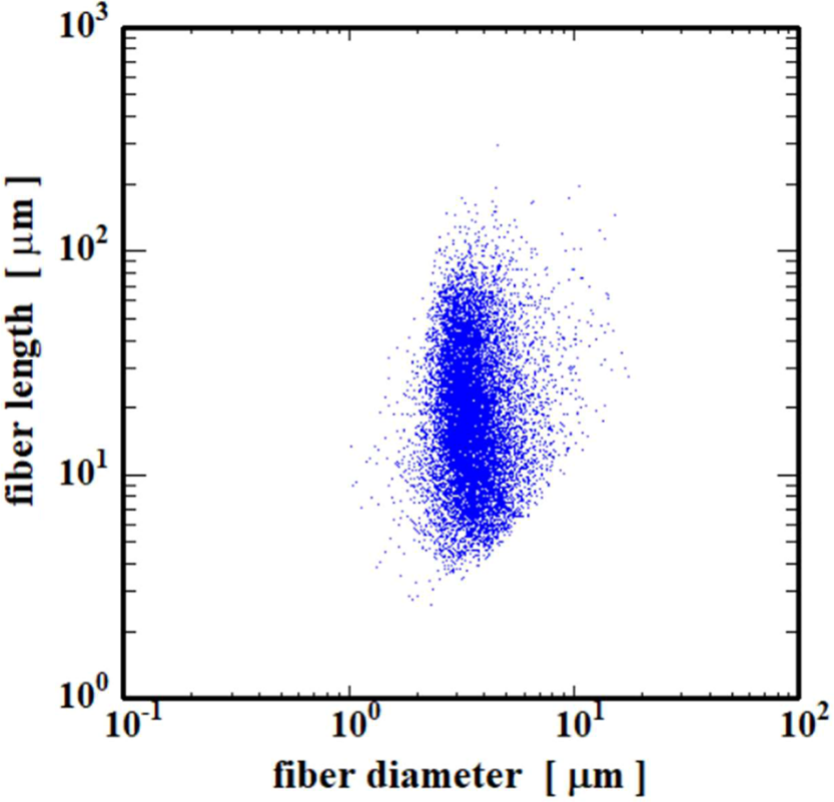
Diameter and length of CNFs calculated from dynamic image analysis.

### 3.2 Viscoelastic Behaviors of CNF Suspensions

Figure 4 shows the results of dynamic viscoelastic measurements of the CNF suspension. Figure 4a shows the strain sweep curve measured with the angular frequency fixed at 10 rad/s. Both the storage modulus (*G*′) and loss modulus (*G*″) were constant at small strains up to a strain amplitude γ_0_ = 2.5 × 10^–2^ (2.5%), and this value was judged to be the linear viscoelastic limit. In the linear region, *G*′ was greater than *G*″. Both components of the dynamic moduli decrease with increasing γ_0_, and eventually, the values of *G*′ and *G*″ intersect at a yield strain γ_*y*_ ≈ 0.2. At larger applied strains γ_0_ ≥ 0.2, the viscous loss modulus exceeds the elastic storage modulus, and the material flows like a viscoelastic liquid. Next, the strain amplitude was fixed at γ_0_ = 1% (within the linear viscoelastic region), and a frequency sweep test was performed (Figure 4b). The storage modulus *G*′ is greater than *G*″ over the entire measured frequency region, and since both elastic moduli are almost independent of the frequency, the material can be viewed as an elasticity-dominated colloidal gel close to equilibrium conditions, and the elasticity is attributed to the entanglements of the dispersed nanofibrils.^37^

**Figure 4:**
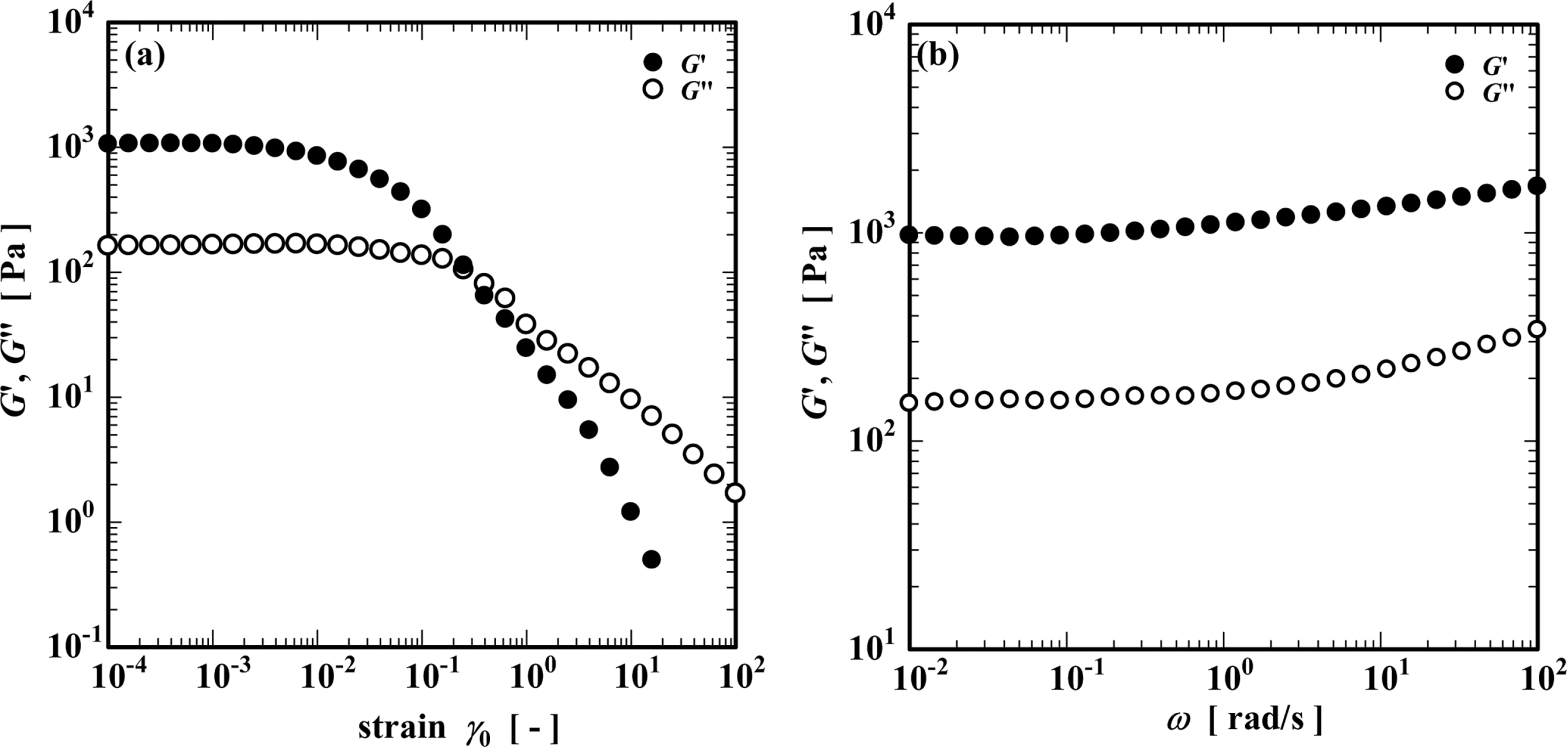
Dynamic viscoelastic behavior of the CNF suspension. (a) Strain sweep at frequency, ω = 10 rad/s. (b) Angular frequency sweep at a strain amplitude of γ_0_ = 0.01(1%).

In addition, when the stress (σ) and strain γ (Figure 5) amplitudes determined during the strain sweep measurement in Figure 4a are cross-plotted, it is evident that the stress amplitude σ initially increases linearly up to a stress of σ ∼ 10 Pa, but the curve begins to flatten and a plateau region is evident over the range of stress from 40 to 60 Pa. This plateau region was judged to be the apparent yield stress of the gel.^38,39^

**Figure 5:**
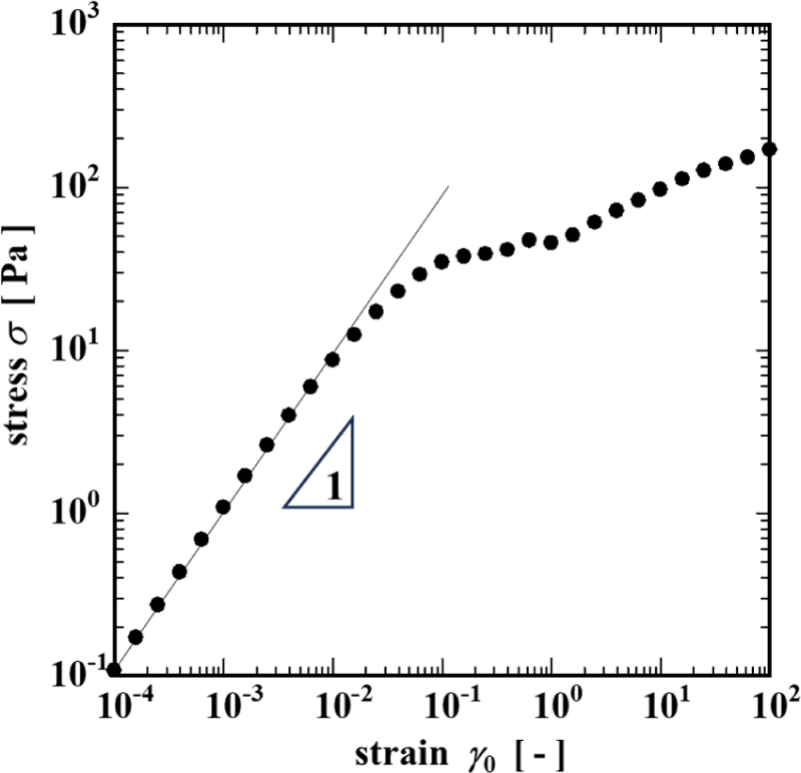
Shear stress–strain amplitude curve of the CNF suspension determined from the strain amplitude sweep.

To evaluate the changes in the internal structure around the yield point, we performed creep tests under a number of different constant stress amplitudes. The applied stresses considered were 10 Pa (below the yield region), 40 and 60 Pa (in the vicinity of the yield region), and finally, 200 Pa, far above the yield transition. We show the creep test results in Figure 6. Under all stresses, strain γ­(*t*) rapidly increases immediately after the measurement began, consistent with an initial elastic response, followed by a slower linear increase at long times, corresponding to viscoplastic flow. It is evident from the ordinate scales in Figure 6 that a 4-fold increase in the applied stress (from 10 to 40 Pa) results in a very large increase in the accumulated deformation, and it is not helpful to replot these measurements in the form of a linear viscoelastic compliance. Instead, we compute the instantaneous rate of shearing $\mathrm{\gamma ̇}\left(t;\sigma_{0}\right)$ , and in Figure 7, we show the evolution in the apparent viscosity $\eta \left(t\right)=\sigma_{0}/\mathrm{\gamma ̇}\left(t\right)$ of the gel with time during the creep tests. When the applied stress was 10 Pa (lower than the yield stress), the viscosity slightly increased over time and reached a very large constant plateau value after about 20 s. When a stress of 40 and 60 Pa (near the yield stress) was applied, the viscosity reaches a much lower plateau (over 3 orders of magnitude smaller) after passing through a local maximum value. The reduction in viscosity is even more pronounced at an applied stress of 60 Pa. In the case of 200 Pa above the yield stress, the viscosity rapidly decreases after shear application before reaching a low constant value of approximately 100 mPa·s after around 30 s.

**Figure 6:**
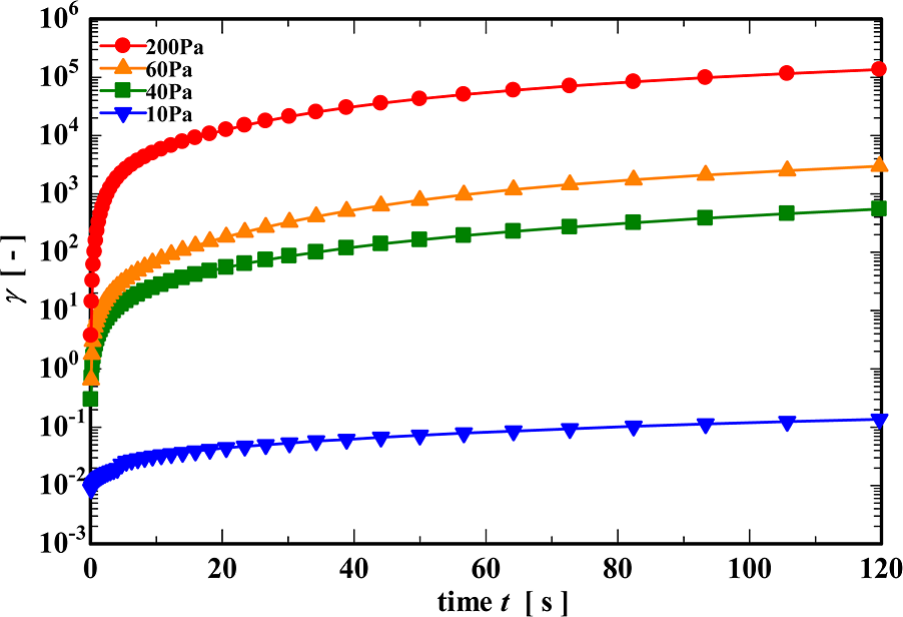
Creep curves of the CNF suspension at stresses below and above the yield transition.

**Figure 7:**
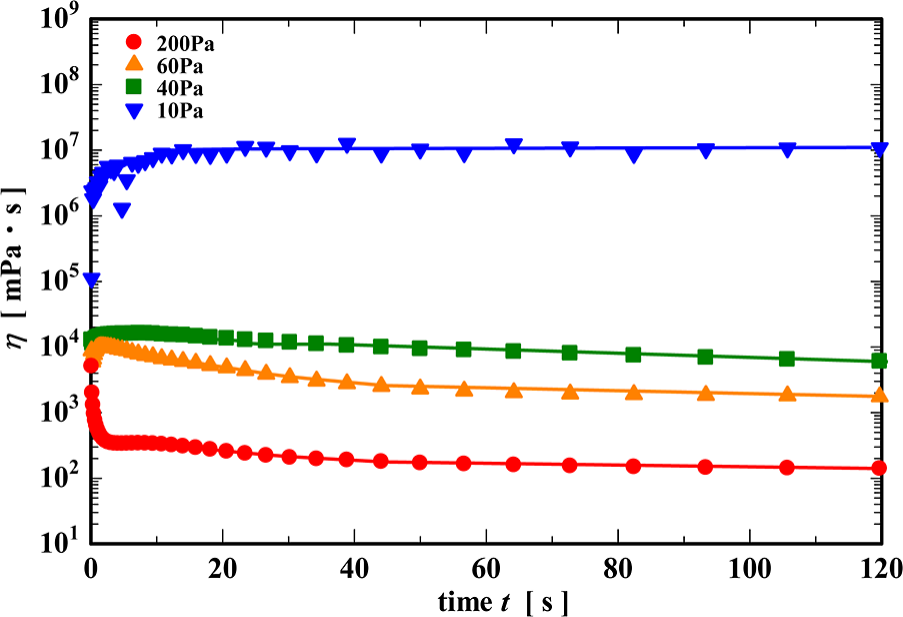
Change in the viscosity of CNF during the creep test.

### 3.3 Rheo-PI Measurements of the CNF Suspension

The incident light emitted from the light source of the device shown in Figure 1 passes through the polarizer (*P*) set at 0° and the quarter-wave plate (*Q*) set at 45° to become circularly polarized light. The circularly polarized light then passes through the sample and emerges with a spatially varying phase difference (Δ) and orientation axis (φ). It then passes through the linear polarizer, which serves as the analyzer with a fast axis orientation of 0°, 45°, 90°, or 135°, and the light intensity distribution is finally measured by the photodetector (details on the calculation of the retardation Δ and orientation axis φ of a sample under shear is provided in the Appendix^40,41^ ). Notably, the raw polarization data are also influenced by the retardation and orientation resulting from the test fixtures, i.e., the glass plate shearing the sample. To remove these influences, we correct the measurements using reference data obtained without the sample, allowing us to isolate the retardance and local microstructural alignment axes originating solely from the sample’s optical anisotropy (integrated through the sample thickness).^29^ As a result, the optical data presented in this paper reflect only the intrinsic properties of the sample, free from external interferences.

Figure 8 shows the dynamic changes in the polarized images of the sample during the creep tests. The image is observed from the underside of the plate, i.e., a planar image in the velocity (flow) direction vs the vorticity direction (1–3 surface). A movie of the time-dependent change in the polarized imaging with time at an applied stress of 40 Pa is shown in Figure S1 of Supporting Information. All the images feature a black mask covering an area with a radius of 12.7 mm from the center of the parallel plate test fixture, which cannot be resolved since light is not transmitted through this region. In the measurement range, low and high retardations are colored dark blue and yellow, respectively. In the rest state (0 s) before the applied stress, the entire sample shows low retardation (dark blue). When a stress of 10 Pa (below the yield stress) was applied, the color change is negligible throughout the test, and low sample retardation persists. When the applied stress increased to 40 Pa (near the yield stress), a localized and linear distribution of retardation was observed initially (20 s). At approximately 60 s, the entire flow field develops a spiral-shaped high-retardation distribution. At a slightly higher stress of 60 Pa (still in the yield region), a spiral-shaped high-retardation region again appears around 20 s and propagates throughout the entire flow field after 40 s. However, after additional shearing for more than 60 s, the retardation in the outermost radial regions of the sample decreased.

**Figure 8:**
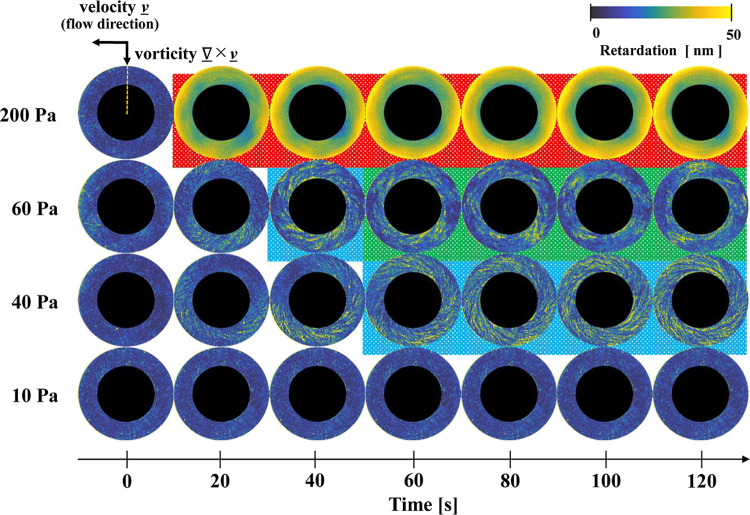
Time-resolved changes in the polarized images of the CNF colloidal suspensions obtained with the Rheo-Iris during creep tests at different applied stresses. The group with light blue shaded background is characterized by a spiral pattern across the entire flow field. The green shaded group is characterized by a low retardation near the outer circumference (where the sample has fully yielded) and a spiral pattern toward the inner part of the plate. The group of images with red shaded background shows high retardation that is azimuthally uniform, but with a radial increase toward the outer edge of the parallel plate fixture, the shear rate is highest.

When a stress of 200 Pa (far exceeding the yield stress) is applied, a high retardation shift from green to yellow appears across the entire measurement area. This shift propagates radially inward from the start of the test and persists until the test ends. Closer examination of the creep test images in Figure 8 shows both spatially uniform and nonuniform (spatially localized) retardation distributions. The nonuniform distributions were further classified into three distinct types, which are shown using light blue, green, and red backgrounds in Figure 8. The images with light blue backgrounds exhibit a spiral pattern across the entire flow field, indicating an inhomogeneous retardation distribution. The group of images with a green-shaded background shows low retardation near the outer circumference at the radial edge of the parallel plate and high retardation in a spiral pattern toward the inner part of the plate. The red-shaded group (corresponding to high stresses and long shearing times) displays an azimuthally uniform retardation distribution but a strong variation with the radial position.

In order to quantify the changes indicated by the polarized images, the average value Δ_avg_ of the retardation over the entire measurement area of each image was calculated, and the change in Δ_avg_ with time is shown in Figure 9. When the applied stress was 10 Pa, Δ_avg_ showed a low retardation of around 10 nm, which hardly changed throughout the duration of the test. The average retardance at an applied stress of 40 Pa gradually increased from 10 to just under 20 nm immediately after the start of the test and eventually became constant after approximately 100 s. In the case of 60 Pa, Δ_avg_ increased from 10 to just under 20 nm until around 30 s, after which it slightly decreased and gradually approached a constant of around 15 nm. The slight decrease in the average retardance Δ_avg_ results from the decreasing retardation near the outer circumference at long times mentioned above. Finally, when a stress of 200 Pa was applied, Δ_avg_ rapidly increased to 30 nm soon after the start of the test as the sample yielded and flowed before gradually approaching a constant value of 35 nm. These results support the qualitative evaluation of the polarized images mentioned above.

**Figure 9:**
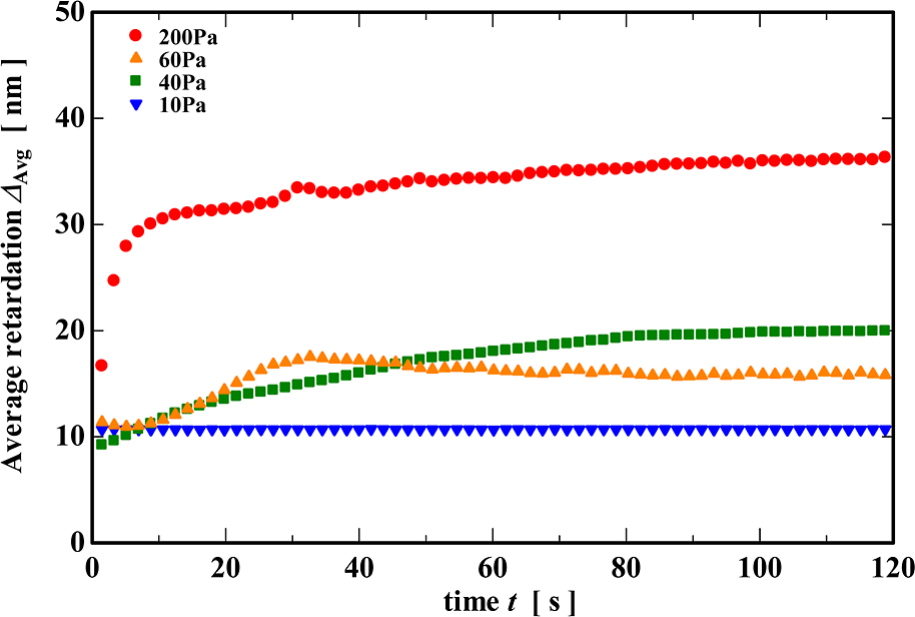
Changes in the average retardation Δ_Avg_ with time during the creep tests.

Next, we evaluated the change in angle Δφ (hereinafter defined between the flow direction and the orientation axis) during the creep tests. Figure 10a shows the definition of Δφ (the local orientation axis of the microstructure φ is indicated by a black bar), and Figure 10b–d shows the spatial distribution of the local orientation axis φ when a stress of 40 and 200 Pa is applied. At 0 s, just before the test started, the orientation axis φ is randomly oriented for both values of the applied stress (Figure 10b,d); however, φ is strongly oriented in the flow direction after 120 s when a shear stress of 200 Pa was applied (Figure 10e). On the other hand, when a lower stress of 40 Pa was applied, the orientation axis was oriented primarily in the vorticity direction (60°–90° with respect to the velocity (flow) direction) (Figure 10c). To summarize, we find that after 120 s, the microstructural orientation φ changes from a disordered to an ordered state and that the degree of orientation differs depending on the applied stress.

**Figure 10:**
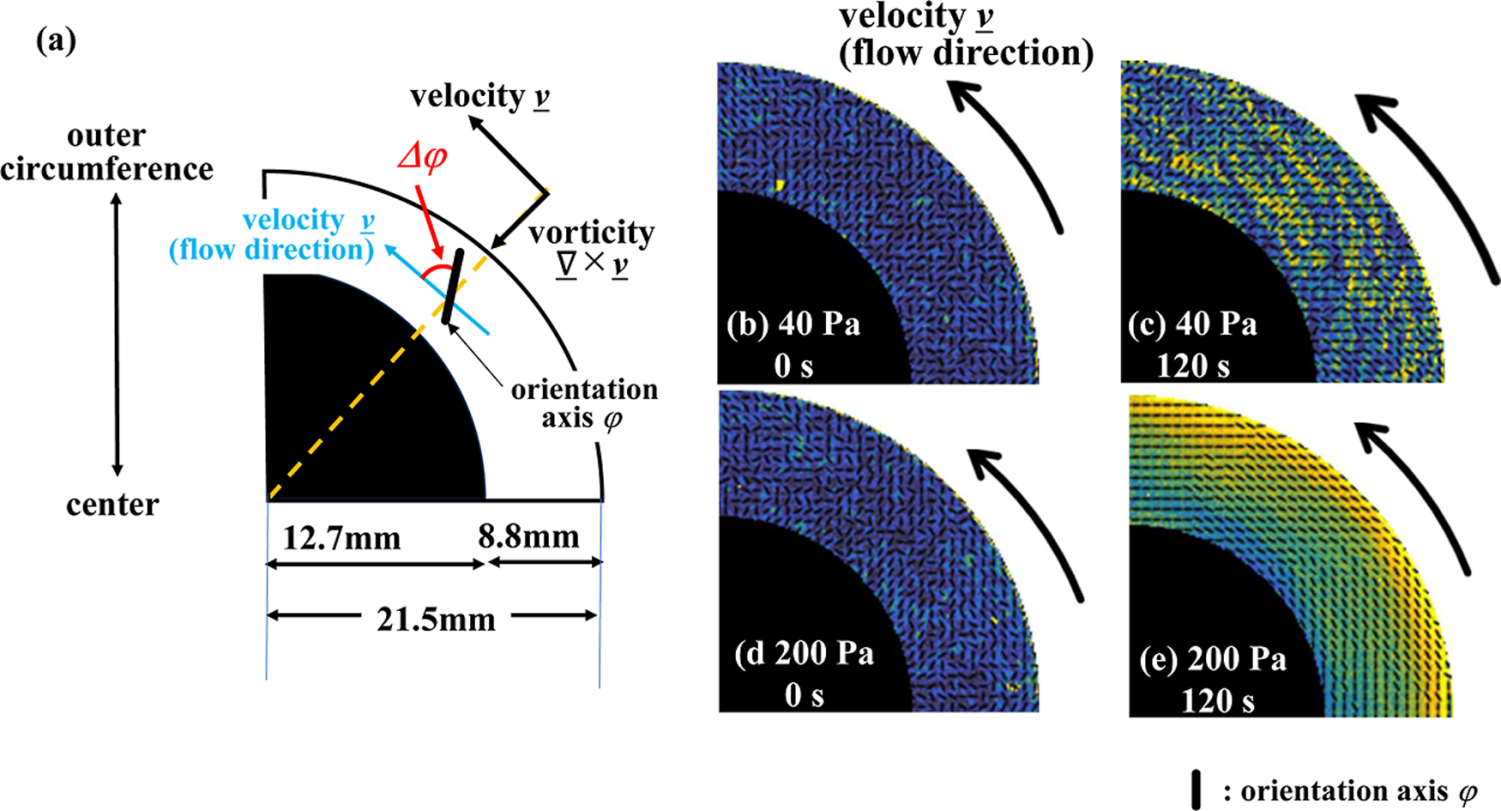
(a) Definition of the angle Δφ and (b–d) change in the orientation axis φ with the applied stress. Orientation axis φ at (b) *t* = 0 s and (c) *t* = 120 s with applied 40 Pa, and (d) *t* = 0 s and (e) *t* = 120 s with applied 200 Pa.

To quantitatively evaluate the orientation axis at an applied stress of 40 Pa and an elapsed time of 120 s, we evaluated the change in the average angle (Δφ_Avg_) on a series of circular rings spanning from close to the center of the plate to the outermost circumference (Figure 11). The average microstructural orientation Δφ_Avg_ on the circumference at a radial distance of 13–15 mm, which is close to the center of the plate, was 85°, close to alignment with the vorticity direction (at 90°, i.e., orthogonal to the flow direction). However, as we move radially outward and approach the outermost circumference, Δφ_Avg_ gradually decreases. From *r* = 19 mm to the outermost circumference, it gradually approaches 60°. These results indicate that the orientation axis, under an applied stress of 40 Pa and a duration of 120 s, is nearly perpendicular (90°) to the vorticity direction at the center and gradually shifts to align more closely with the velocity (flow) direction (0°) in the region of the highest shearing deformation near the outermost edge.

**Figure 11:**
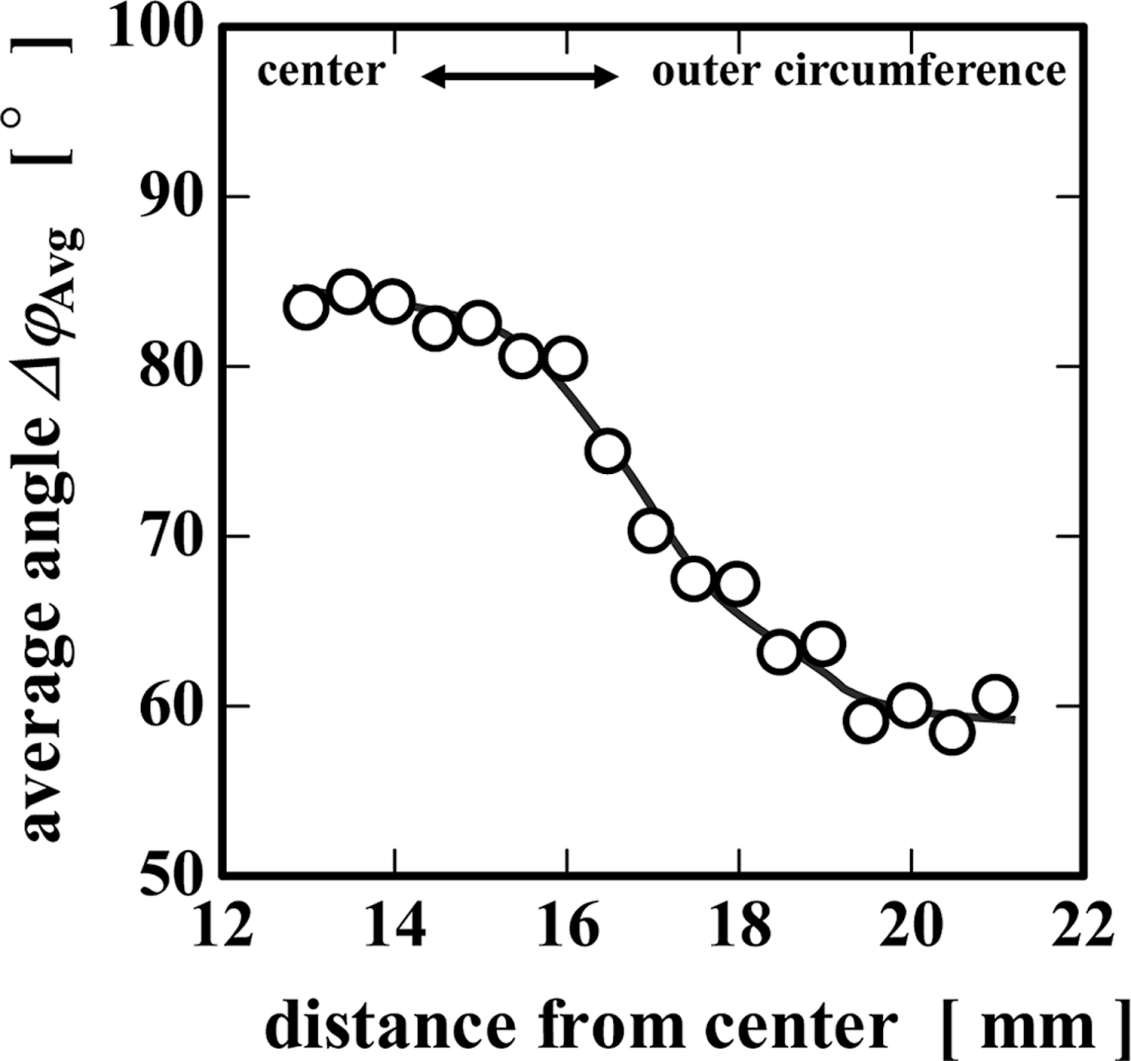
Change in the angle Δφ_Avg_ with applied 40 Pa at 120 s.

Finally, to investigate the spatiotemporal changes in the high retardation region, we converted the polarized images into 8 bit black-and-white images using the open-source software Fiji-ImageJ ver. 1.54p and performed image binarization with a threshold value of 123. Figure 12 shows the binarized forms of the polarized images at 0, 40, 80, and 120 s with an applied stress of 40 Pa shown in Figure 8. The shapes of the local high retardation regions in the contour image after binarization were assumed to be circular or elliptical, and their boundaries were detected using the “Analyze Particles” command in ImageJ. The average values of the major axis (length) and minor axis (width) were then calculated, and finally the change in the aspect ratio, which is the ratio of the major axis to the minor axis, was investigated (Figure 13). It is clear that the aspect ratio of the local highly oriented regions increases with time and gradually approaches a constant value after approximately 100 s. This behavior is similar to the change in Δ_Avg_ shown in Figure 9, and therefore, when the applied stress is close to the yield value, the high retardation regions develop and align along the major axis (length) direction with time.

**Figure 12:**
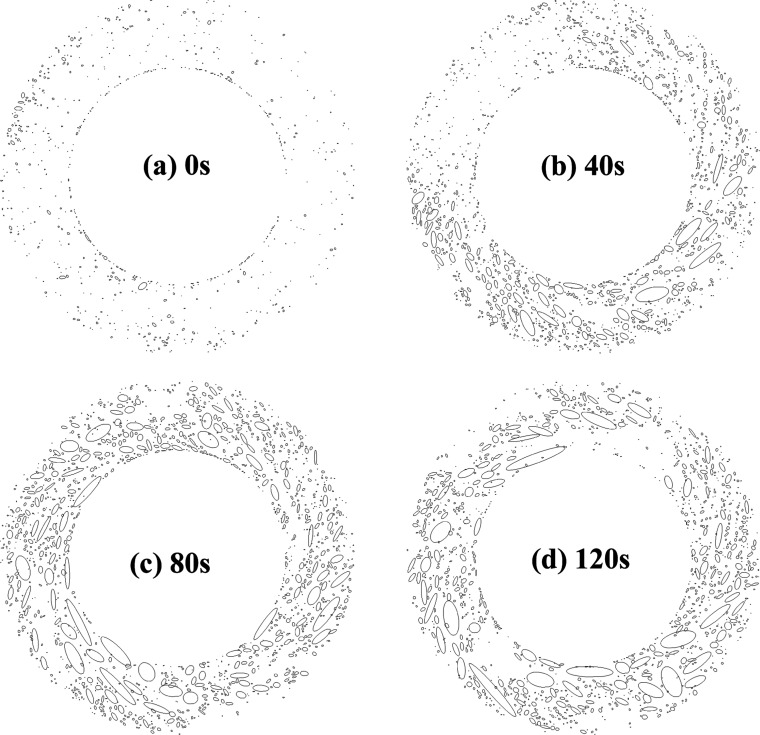
Binarized versions of the polarized images at an applied stress of 40 Pa. (a) 0 s, (b) 40 s, (c) 80 s, (d) 120 s.

**Figure 13:**
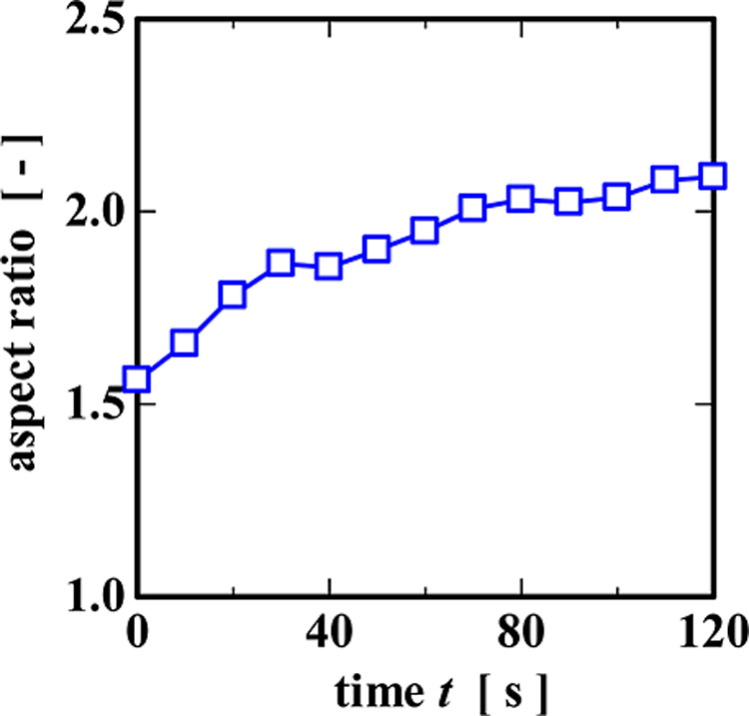
Changes in the aspect ratio of the local highly oriented regions with time at an applied stress of 40 Pa.

### 3.4 Structural Evolution and Orientation Behavior of CNF Fibril Aggregates under Shear Stress

The temporal evolution in the integrated retardation obtained under a constant shearing stress is a measure of the anisotropy in the molecular ordering within the colloidal material, and the orientation axis indicates the average molecular orientation.^42^ In the CNF suspensions, the degree of orientation and direction of the CNF aggregates can be estimated from measurements of the retardation and orientation axis.

Below the yield stress, or immediately after applying a larger shear stress, the retardation was uniformly low across the entire measurement area, and the orientation axis was found to be aligned in a random direction. This implies the initial existence of an isotropic fibril network with extensive fibrillar entanglement when the sample is at rest or under a very small straining deformation.

Above the yield stress transition changes in the retardation and orientation axes appear progressively with time. Spiral-shaped regions with high-order and retardation evolve at an applied stress of 40 Pa. The spatial inhomogeneity of this local retardation distribution indicates that a microstructure with a locally high anisotropy was formed. The time-resolved images show that local regions with both highly ordered and disordered microstructures within the sample coexist. In addition, the orientation axis gradually changes from 90° to 60° as one moves radially outward from close to the center of the plate to the outermost circumference. This corresponds to a progressive evolution from vorticity alignment to alignment in the velocity (flow) direction, and it was found to be linked to the high anisotropy of the local spiral shape. When a large shear stress is applied to the sample, the interactions among the CNF aggregates and/or fibrils that are strongly aligned in the flow direction are progressively disrupted, and spatial inhomogeneities propagate from the regions of highest-deformation near the outer edge toward the center, causing the shear-aligned microstructure to gradually collapse. Ultimately, only the aligned microstructure in the vorticity direction remains, resulting in regions of high retardation in the form of a spiral. In other words, the optical retardation in the gel that develops when a shear stress is applied in the vicinity of the yield region is thought to be caused by the spatially nonuniform arrangement and orientation of multiple CNF aggregates connected in a spiral-like structure in the vorticity direction. In addition, the local orientation axis is not completely orthogonal to the flow direction, and the angle decreases toward the outermost circumference of the plate. This occurs because the accumulated strain generated by the shearing motion of the parallel plate increases radially outward, and the shear rate is also greater at the outer circumference than near the center, so the CNF aggregates near the outer edge of the plate are more quickly oriented in the flow direction. Such spiral polarized images were not observed in a previous study.^29^ The CNFs used in the previous study were of the TEMPO-oxidized type, with a fibril thickness of several nanometers and a length of less than 1 μm,^43^ making them very thin and short compared to those used in this study. Although it wa not stated explicitly in this previous paper, clear spiral-shaped images were not observed for CNFs with short fibril lengths produced by a mechanical fibrillation treatment. The concentration and length of the CNF fibrils in the suspension are probably a major factor, and it is thought that an aligned microstructure perpendicular to the flow direction is unlikely to form unless the fibrils exceed a certain length and entangle.

When a stress of 60 Pa was applied, a spiral-shaped retardation pattern with high orientational order similar to that observed at an applied stress of 40 Pa initially appeared; however, the retardation near the outer edge of the plate decreased with time during the shearing. This possibly occurs because the CNF fibrils or aggregates that are oriented perpendicular to the flow direction become smaller and ultimately collapse due to greater accumulated strain and higher local shear rates.

Finally, when a stress of 200 Pa, far exceeding the yield stress, was applied, the retardation increased across the entire measurement area after 20 s, and the increase in the retardation was localized toward the center, where the local shear rate is small. In addition, the orientation axis was oriented in the flow direction throughout the entire area. Based on these results, it is thought that the “log-rolling” states^23–25^ corresponding to the vorticity-aligned microstructure that forms during the yielding transition collapses into progressively smaller aggregated clusters, and that these structural units are then oriented in the flow direction by the strong shearing flow. Therefore, it can be inferred that the higher average retardation that we observe at the higher shear stress reflects an integral average of the spatially nonuniform oriented birefringence resulting from the collective orientation of many smaller individual CNF aggregate units aligned in the flow direction rather than larger macroscopic structures formed by multiple CNF aggregates. In addition, since the shear rate is higher at the periphery than at the center, these smaller CNF aggregate units are expected to be more significantly oriented in the flow direction near the outer circumference.

Figure 14 shows a conceptual diagram summarizing the results of this studiy. When the sample is at rest or under very small strain deformation, the CNF fibrils form an isotropic percolated network structure with extensive entanglement (Figure 14a). With an increase in the applied stress, the percolated network structure of the dispersed CNF aggregates becomes unstable, and spatial inhomogeneities gradually nucleate and propagate from the outer edge (where the accumulated strain is largest) to the center (Figure 14b). When a stress value close to the yield stress is applied, the sample deformation increases rapidly, and the inhomogeneities between the CNF aggregates propagate further, destroying the percolated network structure, and “log-rolling” states of aggregated CNF aligned in the vorticity direction are formed, resulting in the appearance of the local anisotropy in the oriented birefringence (Figure 14c). Subsequently, when a larger stress exceeding the yield stress is applied, these log-rolling states are disrupted, the alignment in the vorticity direction collapses, and the smaller CNF aggregates elongate and orient in the flow direction due to the strong shear flow, resulting in the anisotropy that develops from the spatially uniform oriented birefringence (Figure 14d). Therefore, this new imaging methodology has revealed for the first time that in the yielding transition of a viscoelastic colloidal gel, the spatial orientation distribution differs significantly depending on the applied stress.

**Figure 14:**
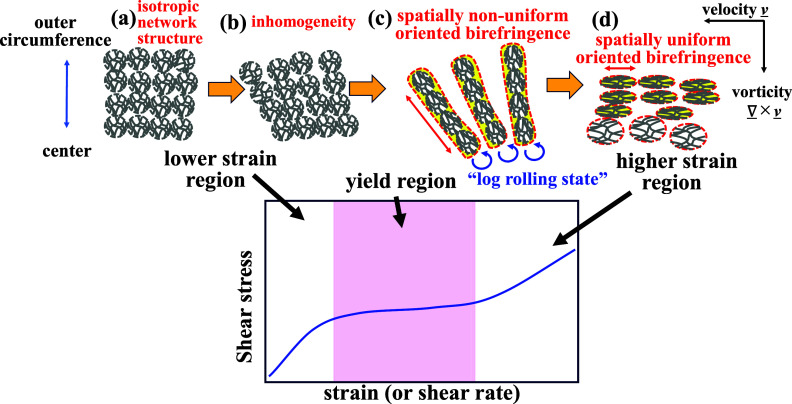
Schematic diagram of the changes in the internal structure of the CNF suspension with the applied stress.

The “Rheo-Iris” allows us to evaluate in real time the spatiotemporal changes in internal mesoscopic heterogeneities and microstructural alignment of a complex gelled material when an external shear stress is applied, which is useful for elucidating the fundamental mechanisms associated with yielding and subsequent flow. The optical configuration of the present device measures the retardation in the 1–3 plane (the velocity–vorticity plane). In the future, we would like to further develop the available test fixtures using a coaxial double cylinder to enable imaging of the retardation in the 1–2 plane (i.e., in the velocity–velocity gradient plane).

## 4 Conclusions

We performed polarized light imaging measurements of CNF suspensions produced by a mechanical fibrillation treatment and evaluated in real time the spatiotemporal changes in the internal microstructure of these elastoviscoplastic colloidal suspensions under different levels of shear stress.

Based on the frequency sweep measurement of the CNF suspension, it was found that the storage modulus *G*′ was greater than the loss modulus *G*″ over the entire measurement frequency range and that both moduli were almost independent of the angular frequency, indicating that this colloidal suspension is in an elastic gel-state under equilibrium conditions. It was also speculated that this elasticity arises from the entanglement of the long nanofibrils.

During creep tests of the CNF suspension, there is initially a large (elastic) strain response, followed by the onset and development of steady creeping flow. The polarized images in the velocity–vorticity plane show three characteristic patterns. Below the yield stress, or immediately after the initial application of a larger stress, the retardation exhibits a uniformly low value across the entire measurement area, and the orientation axis is random and disordered. In other words, it is thought that the elastic deformation of the CNF suspension initially retains an isotropic network structure with extensive entanglements. When a stress of 40–60 Pa near the yield stress is applied, spiral-shaped regions of high retardation appear, and the orientation axis changes from disordered to vorticity-aligned. This is consistent with the CNF aggregates forming log-rolling meso-structures aligned in the vorticity direction, and anisotropy derived from the spatially nonuniform oriented birefringence appears. Finally, when the applied stress is far above the yield stress, the retardation increases throughout the entire measurement area, and the local microstructural orientation axis φ becomes strongly oriented in the flow direction. Thus, it can be considered that the log-rolling structures, which initially develop in the vorticity direction during the yield transition, are disrupted, and the smaller CNF aggregates are elongated and oriented in the flow direction, finally expressing the anisotropy derived from the spatially uniform oriented birefringence.

These new insights into the complexities of the yield transition in an elastoviscoplastic gel strongly suggest that the rheo-polarized imaging technique (“Rheo-Iris”) coupled with direct time-resolved mechanical measurements of the stress/strain/deformation rate relationship in the sheared sample will shine new light on the relationships between the nonlinear rheology and local microstructural evolution of a wide range of complex fluids, not just attractive colloidal suspensions.

## Supplementary Material



## Data Availability

All data generated or analyzed during this study are included in this published article and its Supporting Information files. There is no code availability for software application or custom code.

## Notes

Changes in the strain and polarized images in the creep test (AVI)

## References

[1] Thwala J. M., Goodwin J. W., Mills P. D. (2008). Viscoelastic and shear viscosity
studies of colloidal silica particles dispersed in monoethylene glycol
(MEG), diethylene glycol (DEG), and dodecane stabilized by dodecyl
hexaethylene glycol monoether­(C_12_E_6_). Langmuir.

[2] Nakamura H., Makino S., Ishii M. (2021). Effects of
electrostatic interaction
on rheological behavior and microstructure of concentrated colloidal
suspensions. Colloids Surf., A.

[3] de
Oliveira I. S. S., den Otter W. K., Brielsa W. J. (2012). The origin of flow-induced
alignment of spherical colloids in shear-thinning viscoelastic fluids. J. Chem. Phys..

[4] Loon S. V., Fransaer J., Clasen C., Vermant J. (2014). String formation in
sheared suspensions in rheologically complex media: The essential
role of shear thinning. J. Rheol..

[5] Denn M. M., Morris J. F., Bonn D. (2018). Shear thickening in concentrated
suspensions of smooth spheres in Newtonian suspending fluids. Soft Matter.

[6] Bourrianne P., Niggel V., Polly G., Divoux T., McKinley G. H. (2022). Tuning
the shear thickening of suspensions through surface roughness and
physico-chemical interactions. Phys. Rev. Res..

[7] Prabhu T. A., Singh A. (2022). Rheology and microstructure
of discontinuous shear thickening suspensions. J. Rheol..

[8] Shibayama M., Kawada H., Kume T., Matsunaga T., Iwai H., Sano T., Osaka N., Miyazaki S., Okabe S., Endo H. (2007). In situ small-angle
neutron scattering
and rheological measurements of shear-induced gelation. J. Chem. Phys..

[9] Kawasaki S., Kobayashi M. (2018). Affirmation
of the effect of pH on shake-gel and shear
thickening of a mixed suspension of polyethylene oxide and silica
nanoparticles. Colloids Surf., A.

[10] Yamagata Y., Miyamoto K. (2021). Gel formation and its
relaxation mechanism of shear-induced
aqueous suspensions comprised of bentonite and heptaethylene oleyl
ether. Colloids Surf., A.

[11] Banerjee D., Lew J. H., Luckham P. F. (2024). On the rheological properties of
silica- polyethylene oxide dispersions: Shake gels I the effect of
polymer concentration and temperature. Colloids
Surf., A.

[12] Saarikoski E., Saarinen T., Salmela J., Seppälä J. (2012). Flocculated
flow of microfibrillated cellulose water suspensions: an imaging approach
for characterization of rheological behaviour. Cellulose.

[13] Karppinen A., Saarinen T., Salmela J., Laukkanen A., Nuopponen Ma., Seppälä J. (2012). Flocculation
of microfibrillated
cellulose in shear flow. Cellulose.

[14] Frank M., Anderson D., Weeks E. R., Morris J. F. (2003). Particle migration
in pressure-driven flow of a Brownian suspension. J. Fluid Mech..

[15] Conrad J. C., Lewis J. A. (2008). Structure of colloidal
gels during microchannel flow. Langmuir.

[16] Cheng X., McCoy J. H., Israelachvili J. N., Cohen I. (2011). Imaging the microscopic
structure of shear thinning and thickening colloidal suspensions. Science.

[17] Takeda M., Matsunaga T., Nishida T., Endo H., Takahashi T., Shibayama M. (2010). Rheo-SANS studies on shear thickening in clay-poly­(ethylene
oxide) mixed solutions. Macromolecules.

[18] Akada K., Okubo S., Yamada T., Tokuda K., Yamaguchi K., Uemura S., Onoki T., Tejima S., Kobayashi M., Fujita J. (2023). Anisotropic flocculation in shear thickening colloid-polymer
suspension via simultaneous observation of rheology and X-ray scattering. Colloids Surf., A.

[19] Pignon F., Magnin A., Piau J.-M. (1997). Butterfly
light scattering pattern
and rheology of a sheared thixotropic clay gel. Phys. Rev. Lett..

[20] Belzung B., Lequeux F., Vermant J., Mewis J. (2000). Flow-induced anisotropy
in mixtures of associative polymers and latex particles. J. Colloids Int. Sci..

[21] Lee J., Jiang Z., Wang J., Sandy A. R., Narayanan, Lin S. X.-M. (2018). Unraveling the
role of order-to-disorder transition in shear thickening suspensions. Phys. Rev. Lett..

[22] Krzysko A. J., Nakouzi E., Zhang X., Graham T. R., Rosso K. M., Schenter G. K., Ilavsky J., Kuzmenko I., Frith M. G., Ivory C. F., Clark S. B., Weston J. S., Weigandt K. M., De Yoreo J. J., Chun J., Anovitz L. M. (2020). Correlating inter-particle
forces and particle shape to shear-induced aggregation/fragmentation
and rheology for dilute anisotropic particle suspensions: A complementary
study via capillary rheometry and in-situ small and ultra-small angle
X-ray scattering. J. Colloid Interface Sci..

[23] Lin-Gibson S., Pathak J. A., Grulke E. A., Wang H., Hobbie E. K. (2004). Elastic
Flow Instability in Nanotube Suspensions. Phys.
Rev. Lett..

[24] Varga Z., Grenard V., Pecorario S., Taberlet N., Dolique V., Manneville S., Divoux T., McKinley G. H., Swan J. W. (2019). Hydrodynamics
control shear-induced pattern formation in attractive suspensions. Proc. Natl. Acad. Sci. U.S.A..

[25] Negi A. S., Osuji C. O. (2009). New insights on fumed colloidal rheology – shear
thickening and vorticity-aligned structures in flocculating dispersions. Rheol. Acta.

[26] Potanin A. A. (1993). On the
computer simulation of the deformation and breakup of colloidal aggregates
in shear flow. J. Colloid Inf. Sci..

[27] Potanin A. A., De Rooij R., Van den Ende D., Mellema J. (1995). Microrheological modeling
of weakly aggregated dispersions. J. Chem. Phys..

[28] Hoekstra H., Mewis J., Narayanan T., Vermant J. (2005). Multi length scale
analysis of the microstructure in sticky sphere dispersions during
shear flow. Langmuir.

[29] Sato T., Yamagata Y., Sato Y., Onuma T., Miyamoto K., Takahashi T. (2024). Two-dimensional rheo-optical measurement system to
study dynamics and structure of complex fluids. Appl. Rheol..

[30] Glatter O. (1980). Determination
of particle-size distribution functions from small-angle scattering
data by means of the indirect transformation method. J. Appl. Crystallogr..

[31] Glatter O. (1980). Evaluation
of small-angle scattering data from lamellar and cylindrical particles
by the indirect transformation method. J. Appl.
Crystallogr..

[32] Brunner-Popela J., Glatter O. (1997). Small-angle scattering
of interacting particles. I.
Basic principles of a global evaluation technique. J. Appl. Crystallogr..

[33] Isogai A. (2013). Wood nanocelluloses:
fundamentals and applications as new bio-based nanomaterials. J. Wood Sci..

[34] Lunewski J., Schmidt E. (2024). Application of dynamic
image analysis to the optical
characterization of fibrous bulk material. Particuology.

[35] Nakamura T., Manose H., Hatanaka Y., Uchiyama H., Tozuka Y., Kadota K. (2025). Eco-friendly dry melt
coating of tannic acid particles
with glycerol monostearate and amino methacrylate copolymer for effective
taste masking. ACS Food Sci. Technol..

[36] Yamagata Y., Niinobe S., Suga K., Nakano Y., Miyamoto K. (2022). Rheological
and rheo-optical behaviors of nanocellulose suspensions containing
unfibrillated fibers. Cellulose.

[37] Yamagata Y., Takasaki Y., Miyamoto K. (2024). Rheo-impedance
behavior of cellulose
nanofibers produced by mechanical processing. Cellulose.

[38] Dinkgreve M., Paredes J., Denn M. M., Bonn D. (2016). On different ways of
measuring “the” yield stress. J. Non-Newtonian Fluid Mech..

[39] Homma I., Sato Y., Takahashi T., Noda K., Sogabe A. (2018). A study on
yield behavior and stress relaxation of α-gels. J. Soc. Rheol., Jpn..

[40] Onuma T., Otani Y. (2014). A development of two-dimensional
birefringence distribution measurement
system with a sampling rate of 1.3 MHz. Opt.
Commun..

[41] Nakamine K., Yokoyama Y., Worby W. K. A., Muto M., Tagawa Y. (2024). Flow birefringence
of cellulose nanocrystal suspensions in three-dimensional flow fields:
revisiting the stress-optic law. Cellulose.

[42] Ghanbari R., Terry A., Wojno S., Bek M., Sekar K., Sonker A. K., Nygård K., Ghai V., Bianco S., Liebi M., Matic A., Westman G., Nypelö T., Kádár R. (2025). Propagation
of orientation across lengthscales in sheared
self-assembling hierarchical suspensions via Rheo-PLI-SAXS. Adv. Sci..

[43] Yamagata Y., Suga K., Nakano Y., Takasaki Y., Miyamoto K. (2020). Aspect ratio
of tempo-oxidized nanocellulose and rheological analysis of aqueous
suspensions. J. Soc. Rheol., Jpn..

